# Rhabdomyolysis or Severe Acute Hepatitis Associated with the Use of Red Yeast Rice Extracts: an Update from the Adverse Event Reporting Systems

**DOI:** 10.1007/s11883-023-01157-4

**Published:** 2023-10-13

**Authors:** Maciej Banach, Giuseppe Danilo Norata

**Affiliations:** 1 Department of Preventive Cardiology and Lipidology, Medical University of Lodz (MUL), Lodz, Poland; 2 Department of Pharmacological and Biomolecular Sciences, University of Milan, Milan, Italy

**Keywords:** Monacolin K, Red yeast rice, Lipid-lowering, LDL-C, Rhabdomyolysis, Acute liver injury

## Abstract

**Purpose of Review:**

Elevated plasma levels of low-density lipoprotein cholesterol (LDL-C) are a major risk factor for atherosclerotic cardiovascular disease (ASCVD), and lowering LDL-C reduces the risk of cardiovascular adverse events. Among natural approaches known for their lipid-lowering properties, red yeast rice (RYR) has a cholesterol-lowering effect due to the presence of bioactive components (monacolins) that act by inhibiting the activity of 3-hydroxy-3-methylglutaryl coenzyme A (HMG-CoA) reductase. In August 2018, the European Food Safety Authority (EFSA) concluded in its assessment of the use of RYR (further amended in June 2022) that monacolins from RYR raise significant safety concerns when used as a food supplement at a dose of 10 mg/day. In particular, individual cases of serious adverse effects of monacolins from RYR have been reported at intakes as low as 3 mg/day. The EFSA Panel pointed out several uncertainties regarding the available data.

**Recent Findings:**

We conducted an in-depth and updated analysis of the serious adverse events, with a focus on rhabdomyolysis and acute hepatitis, associated with the consumption of RYR. An analysis of the Food and Drug Administration reporting systems revealed a very small number of cases of rhabdomyolysis or severe acute hepatitis associated with RYR use. In addition, only a few case reports of these serious adverse events associated with RYR use have been published.

**Summary:**

Based on data from adverse event reporting systems and available case reports, the occurrence of rhabdomyolysis or severe acute hepatitis that could be associated with the use of RYR appears to be extremely rare compared to the occurrence with statins, which is rare to common.

## Introduction

Lowering circulating levels of low-density lipoprotein cholesterol (LDL-C) is the mainstay for the prevention of atherosclerotic cardiovascular disease (ASCVD) [1, 2]. Since the introduction of statins, many randomised clinical trials have clearly demonstrated their efficacy and safety [3, 4]. However, some patients experience side effects that may affect adherence to therapy [5]. Among statin-related side effects, muscle symptoms and elevations in liver enzymes are the most common [6]. Furthermore, many patients are unwilling to take statins, preferring “natural products” known for their lipid-lowering properties [7]. Among these products, read yeast rice (RYR) has received much attention because it has been shown to have cholesterol-lowering properties [8•, 9], so it is recommended as a dietary supplement for the treatment of hypercholesterolaemia in the current ESC/EAS guidelines for the management of dyslipidaemias [2].

The main active ingredient in RYR is monacolin K, a natural statin produced during the fermentation of rice with *Monascus purpureus*. Monacolin K is not the only component produced during the fermentation process which instead generates other monacolins, pigments and γ-aminobutyric acid [10], as well as also citrinin, a mycotoxin that is nephrotoxic, teratogenic and genotoxic [11]. For this reason, RYR extracts are tested for the absence of potential contaminants and are part of dietary supplements able to lower plasma cholesterol levels.

Monacolin K is structurally identical to lovastatin; both are rapidly converted in vivo from their lactone to an identical hydroxy acid form, the latter being responsible for inhibiting 3-hydroxy-3 methylglutaryl coenzyme A (HMG-CoA) reductase, the key enzyme involved in the biosynthesis of cholesterol [12]. In RYR, monacolin K is present as a lactone and as a hydroxy acid, the ratio depending on the pH [12]. Lovastatin, conversely, is administered in the lactone form and must be converted to the active hydroxy acid form [12].

Lovastatin is a highly lipophilic compound with poor oral bioavailability (<5%), which is also due to extensive hepatic and intestinal metabolism and transmembrane efflux via P-glycoprotein [13]. However, bioavailability can increase significantly (30–50%) when administered with a standard meal. This effect is related to the involvement of the cytochrome P450 (CYP) 3A4 isoform in the metabolism of lovastatin; co-administration of drugs or food components that inhibit this enzyme increases circulating levels of lovastatin and thus the risk of adverse events [13]. Since monacolin K is identical to lovastatin, such interactions are probably relevant also for monacolin K. However, oral bioavailability of monacolin K in RYR supplements may differ from that of lovastatin: extracts of different RYR products have been shown to inhibit CYP3A4 and/or P-glycoprotein more effectively than pure lovastatin at the same lovastatin content [14]. It is noteworthy that 5–6 mg of monacolin has a comparable cholesterol-lowering effect to 20–40 mg of pure lovastatin, which is probably due to both higher oral bioavailability and synergistic effects of other RYR components [15]. At a dose of 3 mg/day, monacolin K lowered total cholesterol by 11.2% and LDL-C by 14.8% [16].

Although RYR products are generally very well tolerated, consumption of RYR products has been associated, in some available data, mainly individual case reports, with the occurrence of serious adverse events, such as rhabdomyolysis or hepatitis. In August 2018, the European Food Safety Authority (EFSA) adopted a scientific opinion on the safety of monacolins in RYR [17]. The Authority considered that monacolin K in the lactone form is identical to lovastatin and that, based on the available information, the intake of monacolins from red yeast rice via food supplements could result in an estimated exposure to monacolin K in the therapeutic dose range of lovastatin [17]. The available information on adverse effects reported in individuals exposed to RYR was sufficient to conclude that monacolins from RYR raise significant safety concerns at a use level of 10 mg/ day. It also considered that serious adverse effects have been reported at monacolin level of 3 mg/day and that cases of rhabdomyolysis and hepatitis requiring hospitalisation have been recorded [17]. In 2022, based on evidence of adverse health effects associated with the use of monacolins from RYR at levels of 10 mg/day and isolated cases of severe adverse health effects at levels as low as 3 mg/day, the European Commission issued a regulation that RYR products must contain less than 3 mg of monacolins for daily consumption [18••]. The EFSA Panel on Food Additives and Nutrient Sources added to Food (ANS) analysed four sources of case reports (WHO, ANSES, Italian Surveillance System, FDA) and found that the main targets for adverse events were musculoskeletal and connective tissue (29.9–37.2% of cases, including 1–5% of rhabdomyolysis), followed by the liver (9–32%) [17].

However, both the largest meta-analysis of 53 RCTs comprising 112 treatment arms, which included 8535 subjects [19], and next post-marketing nutrivigilance data have shown that RYR is very well tolerated, with an incidence of adverse events of any cause of 0.0374% during the first year of red yeast rice use [20••]. Serious adverse events occurred with a frequency of 0.0003%. Based on these frequencies, the incidence of adverse events attributable to red yeast rice use could be defined as very rare to extremely rare (while those attributable to lovastatin are defined as rare to common) [20••]. Additionally, the above-mentioned meta-analysis confirmed that RYR was not associated with the risk of adverse events even in individuals with statin intolerance, and might also significantly reduce the risk of non-musculoskeletal adverse effects [19].

Because of these discrepancies, we aimed at performing an in-depth and updated analysis of the serious adverse events associated with RYR use, focusing on rhabdomyolysis and acute hepatitis.

## Sources of Data

The FDA Adverse Event Reporting System (FAERS) Public Dashboard is a highly interactive, web-based tool that enables the retrieval of FAERS data in a user-friendly manner to obtain information on human adverse events reported to the FDA by the pharmaceutical industry, health care providers and consumers (https://open.fda.gov/data/faers/).

The CFSAN Adverse Event Reporting System (CAERS) is a database that contains information on adverse events and product complaints submitted to the FDA for foods, dietary supplements and cosmetics (https://open.fda.gov/data/caers/). The database contains data reported by consumers and health care practitioners, data voluntarily reported by industry and data from mandatory reports from dietary supplement industry as of January 2004. The reports in CAERS are evaluated by clinical reviewers in the Centre for Food Safety and Applied Nutrition (CFSAN) to monitor the safety of consumer products. If a potential safety risk is identified in CAERS, further evaluation is conducted.

Being the severe side effects very rare, published case reports retrieved from the literature are limited but are presented to complement the discussion of the topic.

## Results

### Rhabdomyolysis Related to the Use of RYR

Rhabdomyolysis is a potentially life-threatening condition characterised by destruction of skeletal muscle with subsequent release of intracellular enzymes into the bloodstream, leading to systemic complications, the most common being acute renal injury [21]. The actual incidence of rhabdomyolysis is uncertain, mainly because many mild (i.e. oligo-symptomatic or asymptomatic) cases are likely to go unrecognised and it is often difficult to confirm causality between therapy and symptoms.

Rhabdomyolysis can have either physical causes (traumatic injury, muscle hypoxia) or non-physical causes (drugs, toxins and infections) [21]. Several drugs have been associated with an increased incidence of rhabdomyolysis [22]; among them, statin therapy raised concerns due to the severe clinical consequences of this condition and the widespread use of this class of drugs [23].

Monacolin K has the potential to cause rhabdomyolysis. To date, a limited number of cases of rhabdomyolysis have been recorded as attributable to the use of red yeast rice preparations containing monacolin K. In a meta-analysis of 20 studies involving 6663 subjects, no single rhabdomyolysis event was reported in subjects taking RYR preparations containing 4.8 to 24 mg monacolin K per day [24]. In 2017, an analysis of data from the Italian surveillance system showed that among 55 adverse effects attributable to red yeast rice supplements collected between 2002 and 2015, one case of rhabdomyolysis occurred in a subject who has had the same clinical manifestation after taking a statin [25], leading to some causality assessment. In a post-marketing vigilance analysis, 2 cases of rhabdomyolysis were reported in 2 subjects (one requiring hospitalisation); both cases occurred when RYR was added to a statin or following a previous exposure to a statin [20••]. The first case was an elderly woman already taking sertraline and rosuvastatin, who started taking red yeast rice without stopping rosuvastatin; the second case was a person who had experienced rhabdomyolysis in response to simvastatin [20••].

Analysis of data from the FAERS database shows that 43,833 cases of rhabdomyolysis were recorded up to 31 March 2023, of which 4655 were fatal; among these, 4 cases (none fatal) were reported in 4 women taking a red yeast rice supplement (https://open.fda.gov/data/caers/, 2 events occurred in the USA, 2 in Italy) (Table 1). Analysis of data from the CAERS revealed 3 reports of rhabdomyolysis in subjects who had taken red yeast rice (https://open.fda.gov/data/caers/) (Table 1).

**Table 1:** Rhabdomyolysis cases associated with the use of RYR products

FDA reporting systems						
FAERS	Initial FDA received date	Product	Patient age	Sex	Reactions	Case outcome
	27-Sep-2018	*Monascus purpureus*	74 y	Female	Myopathy; rhabdomyolysis	n.a.
	21-Aug-2018	*Monascus purpureus*	77 y	Female	Rhabdomyolysis; myopathy	n.a.
	10-Oct-2018 (event date 12-May-2012)	*Monascus purpureus*	78 y	Female	Myopathy; rhabdomyolysis	n.a.
	07-Sep-2018	*Monascus purpureus*; Sertraline	70 y	Female	Myopathy; food interaction; drug interaction; rhabdomyolysis	n.a.
CAERS	Event date	Product	Patient age	Sex	Reactions	Case outcome
	16-Sep-2005	Red yeast rice 600 mg/d	-	Female	Confusional state; dizziness; fatigue; influenza-like illness; muscular weakness; rhabdomyolysis; tremor	Other outcomes
	23-Feb-2015	Red yeast rice 600 mg/d	78 y	Male	Abasia; arrhythmia; bradycardia; rhabdomyolysis; swelling	Hospitalisation, visited emergency room
	21-Apr-2018	Red yeast rice	61 y	Male	Amnesia; anaemia; ↑blood potassium; bradykinesia; catatonia; claustrophobia; fall; ↑heart rate; mydriasis; ↑platelet count; rhabdomyolysis; ↑weight; ↑white blood cell count	Life-threatening, hospitalisation, other serious or important medical event, visited emergency room
Published case reports						
	Year of publication	Product	Patient age	Sex	Reactions	Case outcome
	2002	Red yeast rice	28 y	Female	Rhabdomyolysis; drug interaction with cyclosporine	n.a.
	2019	Red yeast rice 315 mg/d	65 y	Male	Acute renal deficiency; rhabdomyolysis;	Hospitalisation
	2023	Red yeast rice	50 y	Female	Chest discomfort; myalgia; rhabdomyolysis	Hospitalisation

*n.a.* not available

Only a limited number of case reports were published and are presented below:

1. A 28-year-old renal transplant recipient woman experienced post-transplant hypercholesterolaemia (LDL-C 4.2 mmol/L [162 mg/dL]); diet failed to improve the lipid profile, but the patient refused to start statin therapy. A routine blood test revealed a creatine phosphokinase (CPK) level of 2600 U/L without any symptoms; she stated she had been taking an unapproved herbal preparation containing red yeast rice, ß-sitosterol and garlic bulb for the previous 2 months. After supplement discontinuation, CPK levels dropped [26]. Since the metabolism of statins is affected by cyclosporine (used to prevent transplant rejection), which shares the same CYP3A4 clearance pathway, concurrent therapy with this drug increases the risk of severe adverse effects from statins. In this patient, a drug-herbal interaction probably occurred, resulting in increased serum levels of monacolin K and rhabdomyolysis. Drug-drug interactions cannot be ruled out. Neither the dose nor the type of RYR supplement used was mentioned [26].
2. A 65-year-old healthy man was admitted to the hospital with acute renal failure, hepatitis and rhabdomyolysis [27]. In the 14 days prior to admission, the patient had not taken any drugs or medications other than regular acetylsalicylic acid 75 mg/day and red yeast rice 315 mg/day (with 10 mg monacolin K), supplemented by paracetamol max 3 g/day, ibuprofen 200–400 mg/day and single doses of opioids administered by medical staff. The joint medication chart showed that he should have been taking aspirin and atorvastatin, but he had replaced atorvastatin with red yeast rice 315 mg/day 11 months earlier, assuming that the latter was free of side effects. The patient’s condition improved during hospitalisation without dialysis or major interventions other than fluid therapy and administration of antibiotics and discontinuation of red yeast rice. After 9 days in hospital, he was discharged for outpatient follow-up. It was only two and a half months later that his clinical values had normalised. They suspected side effects of red yeast rice as the cause [27].
3. A 50-year-old woman diagnosed with dyslipidaemia and with a history of hypertension and depression was hospitalised with chest discomfort and generalised myalgia [28]. Blood analysis revealed high levels of CPK, lactate dehydrogenase (LDH) and myoglobin. She had been taking a RYR preparation for 3 days prior to admission. Two weeks after discontinuation of the RYR preparation, the parameters normalised and the clinical course was favourable. The RYR and monacolin K doses are not given in this case report.

### Rhabdomyolysis Related to the Use of Statins

All statins have the potential to cause muscle damage in a dose-dependent manner, although they differ in several properties. Simvastatin, atorvastatin and lovastatin are metabolised by CYP3A4 (the most abundant cytochrome P450 isoform); co-administration with other drugs that inhibit CYP3A4 may result in increased statin blood levels and promote toxicity [23]. Rosuvastatin and fluvastatin are metabolised by the CYP2A9 isoenzyme and therefore carry a lower risk of drug-drug interactions. Pitavastatin has a unique metabolism without using CYP P450 pathway (only in few percentage CYP2A8 and CYP2A9) [29]. In addition, almost all statins are substrates of organic anion transporting polypeptides transporter (OATP1B1), and co-administration of drugs that inhibit OATP1B1 increases plasma levels of statins and possibly the incidence of adverse muscle events.

A retrospective study of 8,610 cases of drug-associated rhabdomyolysis reported to the US Food and Drug Administration (FDA) from 2004 to 2009 found that simvastatin, atorvastatin and rosuvastatin were the most common suspected causes and accounted for 3945 cases (45%) [30]. Extending this search to all drug-related rhabdomyolysis cases available in the FAERS database (from 1992 to 31 March 2023), 14,591 of 43,833 cases were due to statins (https://open.fda.gov/data/faers/). It is noteworthy that 238 cases of rhabdomyolysis likely related to lovastatin use have been recorded in this database to date (https://open.fda.gov/data/faers/). A network meta-analysis estimated the relative risk of statin-associated musculoskeletal symptoms across 24 RCTs and more than 150,000 patients and showed that out of 200 patients receiving high-intensity statin therapy, one patient could experience adverse muscle effects compared with moderate-intensity statin therapy [31]. The incidence of rhabdomyolysis was very low (<0.05%), making statistical comparisons inconclusive. Overall, there was a non-significant increase in the relative risk of rhabdomyolysis between placebo and moderate-intensity therapy [RR=1.39, 95% CI 0.68 to 2.86], a non-significant increase between moderate-intensity and high-intensity therapy [RR=2.45, 95% CI 0.46 to 13.05] and a non-significant decrease between placebo and high-intensity therapy [RR=0.96, 95% CI 0.22 to 4.09].

The best-known case of an association between statin therapy and rhabdomyolysis involves cerivastatin. Cerivastatin, a synthetic powerful statin, was withdrawn from the market worldwide in 2001 because of a greater incidence of rhabdomyolysis cases, including fatal cases, compared to other statins. Four years after its launch, 52 fatal cases of rhabdomyolysis leading to kidney failure and 385 non-fatal cases were reported among ~700,000 users in the USA [32]. Sequencing of *SLCO1B1*, the gene encoding OATP1B1, identified genetic variants associated with a significant reduction in cerivastatin uptake in 122 patients who developed rhabdomyolysis while on cerivastatin. In addition, the screening of 15 drugs commonly taken by patients who experienced rhabdomyolysis showed that most of them inhibited OATP1B1-mediated uptake of cerivastatin [33]. These results suggest that reduced uptake of cerivastatin by OATP1B1 variants or concomitantly administered drugs that act as OATP1B1 inhibitors may decrease the transport and metabolism of cerivastatin and increase the risk for adverse reactions.

Concomitant use of statins and other drugs may significantly increase the incidence of rhabdomyolysis. A meta-analysis comparing the effects of daptomycin, a cyclic lipopeptide antibiotic, given alone or together with a statin, showed that the incidence of daptomycin-related rhabdomyolysis was significantly higher in patients taking a statin [34]. Similar results were reported in another meta-analysis [35]. It must be emphasised, however, that knowledge of the metabolism of concomitant drugs and possible interactions with statins always provides the opportunity to substitute the statin with another (usually a hydrophilic one that spares hepatic metabolism), reduce the statin dose (and/or concomitant therapy) and, depending on baseline cardiovascular risk, add a non-statin therapy that does not use the CYP P450 pathway and is very safe (ezetimibe, bempedoic acid and PCSK9-targeted therapy approach). In this way, we can effectively treat most patients with different types of cancer or hepatitis C virus disease or patients who need immunosuppressive therapy without safety concerns.

### Severe Acute Liver Adverse Events Related to the Use of RYR

Among the adverse events associated with the use of RYR, acute liver injury was suggested of great importance. Mazzanti et al. reported hepatic adverse events in 10 individuals who had taken a red yeast rice supplement, 4 of whom had an increase in liver enzyme levels and 6 had acute hepatitis requiring hospitalisation [25]. A post-marketing analysis of dietary supplements containing red yeast rice showed that hepatic adverse events (including transaminase elevations) occurred in 26 of the 855 adverse events reported (3%), with a frequency of 0.0011% among those exposed to red yeast rice [20••]. Among these, severe hepatitis was diagnosed in 7 patients with causality considered probable in 1 case and possible in 2 cases [20••]. The FAERS database recorded 3 cases of hepatic cytolysis in subjects taking red yeast rice, none of which were fatal (https://open.fda.gov/data/faers/) (Table 2); the CAERS database recorded 1 case of unspecified liver injury in a 69-year-old woman and 1 case of liver failure following red yeast rice use in a 46-year-old man were recorded in https://open.fda.gov/data/caers/ (Table 2). These findings are consistent with a recent meta-analysis of nine studies involving 195,602 participants with chronic viral hepatitis, which found no significant difference in the risk of mortality between statin users and non-users in the overall analysis. The authors showed that the risk of mortality decreased significantly by 39% in statin users who were followed for more than 3 years. In addition, the risk of hepatocellular carcinoma, fibrosis and cirrhosis decreased by 53%, 45% and 41%, respectively, in those taking statins. Although ALT and AST decreased slightly after statin therapy, this reduction was not statistically significant [36].

**Table 2:** Severe hepatic adverse event cases associated with the use of RYR products

FDA reporting systems						
FAERS	Initial FDA received date	Product	Patient age	Sex	Reactions	Case outcome
	14-Aug-2019	*Monascus purpureus*	56 y	Male	Drug Interaction; hepatic cytolysis	n.a.
	26-Mar-2019	*Monascus purpureus*	56 y	Male	Hepatic cytolysis; drug interaction	n.a.
	29-May-2013	*Monascus purpureus*	45 y	Male	Urticaria; hepatic cytolysis	n.a.
CAERS	Event date	Product	Patient age	Sex	Reactions	Case outcome
	04-Apr-2011	Red yeast rice	69	Female	Chromaturia; faeces discoloured; hypercholesterolaemia; jaundice; liver injury	Other serious or important medical event, visited a health care provider
	07-Jan-2017	Red yeast rice	46	Male	Hepatic failure	Life threatening, hospitalisation, disability
Published case reports						
	Year of publication	Product	Patient age	Sex	Reactions	Case outcome
	2008	Red yeast rice 600 mg	62 y	Female	Flu-like symptoms; nausea; vomiting; diarrhoea; chills; daily fever; hepatitis	Hospitalisation
	2009	Red yeast rice (monacolin K 10 mg)	71 y	Female	Fulminant hepatitis with cytolysis	Death
	2019	Red yeast rice 1200 mg	50 y	Female	Acute hepatitis	Hospitalisation
	2019	Red yeast rice 315 mg	65 y	Male	Acute renal deficiency; hepatitis rhabdomyolysis	Hospitalisation

*n.a.* not available

Only a few case reports have been published describing cases of acute hepatitis in subjects taking red yeast rice supplements (Table 2).

1. A 62-year-old woman with no history of liver disease was admitted to the hospital with a 10-week history of flu-like symptoms followed by 1 week of nausea, vomiting, diarrhoea, chills and daily fever after taking two 600 mg capsules of RYR twice a day for approximately 4 months before admission. She had elevated liver enzyme levels; liver biopsy showed moderate acute and chronic lobular inflammation with features consistent with drug-induced hepatitis. The patient improved clinically after she stopped consuming red yeast rice [37].
2. A fatal fulminant hepatitis in a 71-year-old woman likely associated with consumption of a dietary supplement containing 10 mg monacolin K. After 3 months of consumption, biological examinations revealed major abnormalities in the liver, suggestive of acute hepatitis with predominant cytolysis; the situation worsened despite initiation of therapy to treat autoimmune hepatitis suggested by liver biopsy, and the patient died with a final diagnosis of fulminant hepatitis [38].
3. A 64-year-old woman who had not previously taken any medication was hospitalised with acute hepatitis 6 weeks after starting a regiment of red yeast rice (1200 mg/day) to treat hypercholesterolaemia. Liver biopsy results were consistent with acute drug-induced hepatitis. Although alcohol consumption could have contributed to the disease, the acute nature and timing of her disease suggested drug-induced liver injury as the aetiology [39].
4. A 65-year-old healthy man was hospitalised with acute renal failure, hepatitis and rhabdomyolysis after he started taking a red yeast rice supplement (see above for clinical presentation) [27].

### Severe Acute Liver Adverse Events Related to the Use of Statins

Drug-induced liver damage has been also described as a side effect of statins, although it is extremely rare. Elevations in liver enzymes (transaminases), mostly temporary, may occur in up to 3% of patients treated with statins [40]. However, the risk of liver injury with clinical consequences is rather rare, although it may be associated with severe outcomes [41]. In the FAERS database, of the 5516 cases of drug-related acute hepatitis, 257 were related to statin use and only 2 were related to lovastatin use (https://open.fda.gov/data/faers/). Severe liver injury appears to be uncommon with statins and is generally reversible after therapy discontinuation. In a recent clinical investigation, 11 cases of drug-induced liver injury associated with statin use were reported; atorvastatin was most commonly involved in the development of cholestatic liver injury (8 cases) [42], which is consistent with a previous study [41].

## Limitations

We must acknowledge some limitations. First, these adverse event reporting systems accept voluntary reports from consumers, health professionals and manufacturers, as well as mandatory reports from dietary supplement manufacturers. Even if FAERS contains reports about a particular drug or biologic, this does not mean that the drug or biologic caused the adverse event. The FDA does not require that a causal relationship between a product and event be proven, and reports do not always contain enough detail to properly evaluate an event. In addition, the FDA does not receive a report for every adverse event or medication error that occurs with a product. FAERS data is not in itself an indicator of the safety profile of the drug or biologic. Some reports may be duplicates (such as in the case the same report is submitted by a consumer and the manufacturer); some reports do not contain all the required information. The information in the reports has not been verified and there is no certainty that a particular drug caused the adverse event. Similarly, the adverse event reports in CAERS for a product and the total number of adverse event reports for that product reflect only information and do not represent a conclusion by FDA as to whether the product actually caused the adverse events. With any report, there is no certainty that a suspected product caused a reaction. The reported event may also be related to an underlying disease or activity, or the concurrent use of another product, or it may simply have occurred coincidentally at that time. Reports submitted to the FDA vary in terms of the quality and reliability of the information provided. Some reports to the FDA do not necessarily include all relevant data, for example, whether a person also suffered from other diseases or was taking other products or drugs at the same time. The information in these reports has not been scientifically or otherwise tested for a cause-and-effect relationship and cannot be used to estimate the actual incidence or risk.

## Conclusion

EFSA concluded in its assessment of the use of RYR that monacolins from RYR pose a significant safety risk when used as a food supplement at a dose of 10 mg/day, with rhabdomyolysis and severe acute hepatitis being the most serious adverse effects observed. However, nutrivigilance monitoring does not support this conclusion and further ongoing nutrivigilance monitoring and investigation is required to determine the association, if any, between monacolins, rhabdomyolysis and severe acute hepatitis.

An in-depth analysis of the FAERS found that of 43,833 recorded cases of rhabdomyolysis, only 4 cases (none fatal) were associated with RYR use; similarly, of 23,339 reported cases of acute hepatitis/hepatic cytolysis, only 3 cases (none fatal) were associated with RYR use. Similarly, data from the CAERS indicate that only 3 cases of rhabdomyolysis and 2 cases of liver injury (one of which was classified as undefined liver injury) were associated with RYR use.

In summary, the available data suggest that the occurrence of rhabdomyolysis or severe acute hepatitis that could be associated with RYR use is very to extremely rare compared to cases reported to be associated with statins, which are rare to common.
